# Reduced cortical thickness of the paracentral lobule in at-risk mental state individuals with poor 1-year functional outcomes

**DOI:** 10.1038/s41398-021-01516-2

**Published:** 2021-07-14

**Authors:** Daiki Sasabayashi, Yoichiro Takayanagi, Tsutomu Takahashi, Shimako Nishiyama, Yuko Mizukami, Naoyuki Katagiri, Naohisa Tsujino, Takahiro Nemoto, Atsushi Sakuma, Masahiro Katsura, Noriyuki Ohmuro, Naohiro Okada, Mariko Tada, Motomu Suga, Norihide Maikusa, Shinsuke Koike, Atsushi Furuichi, Mikio Kido, Kyo Noguchi, Hidenori Yamasue, Kazunori Matsumoto, Masafumi Mizuno, Kiyoto Kasai, Michio Suzuki

**Affiliations:** 1 Department of Neuropsychiatry, University of Toyama Graduate School of Medicine and Pharmaceutical Sciences, Toyama, Japan; 2 Research Center for Idling Brain Science, University of Toyama, Toyama, Japan; 3 Arisawabashi Hospital, Toyama, Japan; 4 Center for Health Care and Human Sciences, University of Toyama, Toyama, Japan; 5 Department of Neuropsychiatry, Toho University School of Medicine, Tokyo, Japan; 6 Department of Psychiatry, Saiseikai Yokohamashi Tobu Hospital, Kanagawa, Japan; 7 Department of Psychiatry, Tohoku University Hospital, Sendai, Japan; 8 Osaki Citizen Hospital, Miyagi, Japan; 9 Department of Neuropsychiatry, Graduate School of Medicine, The University of Tokyo, Tokyo, Japan; 10 International Research Center for Neurointelligence (WRI-IRCN), UTIAS, The University of Tokyo, Tokyo, Japan; 11 Graduate School of Clinical Psychology, Teikyo Heisei University, Tokyo, Japan; 12 Center for Evolutionary Cognitive Sciences (ECS), Graduate School of Art and Sciences, The University of Tokyo, Tokyo, Japan; 13 Department of Radiology, University of Toyama Graduate School of Medicine and Pharmaceutical Sciences, Toyama, Japan; 14 Department of Psychiatry, Hamamatsu University School of Medicine, Hamamatsu, Japan; 15 Kokoro no Clinic OASIS, Sendai, Japan

**Keywords:** Neuroscience, Prognostic markers

## Abstract

Although widespread cortical thinning centered on the fronto-temporal regions in schizophrenia has been reported, the findings in at-risk mental state (ARMS) patients have been inconsistent. In addition, it remains unclear whether abnormalities of cortical thickness (CT) in ARMS individuals, if present, are related to their functional decline irrespective of future psychosis onset. In this multicenter study in Japan, T1-weighted magnetic resonance imaging was performed at baseline in 107 individuals with ARMS, who were subdivided into resilient (77, good functional outcome) and non-resilient (13, poor functional outcome) groups based on the change in Global Assessment of Functioning scores during 1-year follow-up, and 104 age- and sex-matched healthy controls recruited at four scanning sites. We measured the CT of the entire cortex and performed group comparisons using FreeSurfer software. The relationship between the CT and cognitive functioning was examined in an ARMS subsample (*n* = 70). ARMS individuals as a whole relative to healthy controls exhibited a significantly reduced CT, predominantly in the fronto-temporal regions, which was partly associated with cognitive impairments, and an increased CT in the left parietal and right occipital regions. Compared with resilient ARMS individuals, non-resilient ARMS individuals exhibited a significantly reduced CT of the right paracentral lobule. These findings suggest that ARMS individuals partly share CT abnormalities with patients with overt schizophrenia, potentially representing general vulnerability to psychopathology, and also support the role of cortical thinning in the paracentral lobule as a predictive biomarker for short-term functional decline in the ARMS population.

## Introduction

Current evidence supports that early detection and intervention can lead to less severe negative symptomatology and better functional outcomes in patients with schizophrenia [1, 2], potentially by preventing and/or ameliorating active brain changes at early stages of psychosis [3–5]. However, brain morphological characteristics possibly associated with early neurodevelopmental abnormalities, such as altered sulco-gyral patterns [6–9], may also be related to a lower rate of remission and poor outcomes in schizophrenia. Recent high-risk studies further demonstrated that the majority of individuals with at-risk mental state (ARMS) [10, 11] do not develop psychosis [12] but experience long-term functional impairments [13] due to several factors such as persistent subthreshold symptoms [14], comorbid depression and anxiety [15], and socio-cognitive dysfunction [16]. Whereas the onset of psychosis has been the outcome measure of interest in almost all previous high-risk studies [17], functional impairment has also been gaining attention as another important indicator [18], as it is associated with the development of persistent and recurrent nonpsychotic disorders that are commonly seen in ARMS and require ongoing clinical care [19]. However, neural underpinnings associated with functional outcomes in ARMS individuals remain largely unknown.

Cortical thickness (CT) may be a more sensitive measure of subtle cortical changes than gray matter volume [20, 21]. Schizophrenia patients likely exhibit widespread cortical thinning centered on the fronto-temporal regions [22], where baseline findings at first-episode [23, 24] and/or excessive thinning during the course of illness [25] may partly underlie cognitive impairments [26–28] and poor clinical outcomes [29, 30]. However, it remains unclear whether such CT abnormalities precede the onset of psychosis due to inconsistent findings [31–33] and whether CT findings in ARMS individuals are related to cognitive function and/or functional outcomes. A recent study by de Wit et al. [34] reported the association between widespread cortical thinning at baseline and poor functional outcomes at 6-year follow-up in a relatively small ARMS cohort (*n* = 35), but a potential relationship between the CT abnormalities in ARMS individuals, if present, and their shorter-term outcomes remains largely unknown.

In the present study, we investigated CT abnormalities at baseline and their relevance to functional outcomes at the 1-year follow-up in a relatively large sample of ARMS individuals recruited at four Japanese scanning sites. We also examined the relationship between the CT findings and cognitive functioning in an ARMS subsample. Based on the previous findings in ARMS individuals [31, 34], we predicted widespread CT reduction, especially in ARMS individuals with poor functional outcomes. Based on previous findings in schizophrenia [27, 28], we also predicted that cortical thinning centered on the frontal region is related to specific cognitive impairments in the ARMS population.

## Materials and methods

### Participants

From September 2006 to August 2012, we recruited 107 ARMS individuals at four hospitals with specialized ARMS services [35, 36]: Toyama University Hospital, Toho University Hospital, Tohoku University Hospital, and The University of Tokyo Hospital (Table 1) [37]. A consensus diagnosis of ARMS for all patients was provided by clinical research meetings at each site based on the interview data using the Comprehensive Assessment of At-Risk Mental States (CAARMS) [10] (Toyama and Tohoku) or the Structured Interview for Prodromal Symptoms/the Scale of Prodromal Symptoms [38] (Toho and Tokyo). ARMS individuals were evaluated using the Global Assessment of Functioning (GAF) [39] both at MRI (baseline) and a year after (follow-up), and classified into good (ARMS-resilient [ARMS-R], baseline score ≤ follow-up score, *n* = 77) or poor (ARMS-non-resilient [ARMS-NR], baseline score > follow-up score, *n* = 13) functional outcome subgroups. During the follow-up period (mean = 4.9 years, SD = 2.6 years), 21 ARMS individuals (14 ARMS-R and 3 ARMS-NR individuals) subsequently developed psychosis, whose diagnoses using the Diagnostic and Statistical Manual of Mental Disorders—Fourth Edition (DSM-IV) [40] were schizophrenia (*n* = 14), delusional disorder (*n* = 1), schizophreniform disorder (*n* = 1), brief psychotic disorder (*n* = 1), and psychotic disorder not otherwise specified (*n* = 4). At baseline, a substantial number of ARMS individuals (*n* = 43/107, 40.2%) were using the minimum dosage of antipsychotics for their mental condition (e.g., rapid deterioration or suicidal and harmful risk) according to previous reports [41]. Cognitive function was rated at baseline using Japanese version of the Brief Assessment of Cognition in Schizophrenia (BACS) [42] in a subsample of ARMS individuals (*n* = 70). We used raw scores for the six BACS subdomains: (i) list learning as verbal memory, (ii) digit sequencing task as working memory, (iii) token motor task as motor speed, (iv) category and letter fluency as verbal fluency, (v) symbol coding as attention and processing speed, and (vi) the Tower of London task as executive function. We also recruited 104 age- and sex-matched healthy controls from the community, hospital staff, and university students at each site (Table 1). All participants were physically healthy, and none had a history of serious head injury, neurological illness, substance abuse or dependence (including cannabis use) disorder, or previous psychotic episode that fulfilled the DSM-IV criteria. All subjects were included in our previous study that assessed the subcortical volumes in ARMS individuals [43]. After a complete explanation of the design and purpose of the study, which was approved by the medical ethics committee of each site, written informed consent was received from all participants.

**Table 1:** Characteristics of the study subjects.

	HC	Whole ARMS	ARMS-R	ARMS-NR	HC vs whole ARMS		ARMS-R vs ARMS-NR	
					Test statistic	*P* value	Test statistic	*P* value
Number of subjects (Total), no. of subjects (No. of ARMS-P subjects)	104	107	77 (14)	13 (3)				
Scanning site 1 (Toyama), no. of subjects (No. of ARMS-P subjects)	52	22	16 (4)	2 (1)				
Scanning site 2 (Toho), no. of subjects (No. of ARMS-P subjects)	4	19	12 (2)	1 (0)				
Scanning site 3 (Tohoku), no. of subjects (No. of ARMS-P subjects)	17	35	29 (6)	4 (1)				
Scanning site 4 (Tokyo), no. of subjects (No. of ARMS-P subjects)	31	31	20 (2)	6 (1)				
Follow-up period, years, means (SD)		4.9 (2.6)	5.4 (2.4)	4.0 (2.0)				
Sex, male/female, no.	52/52	49/58	39/38	4/9	*χ*^2^(1) = 0.37	0.54	*χ*^2^(1) = 1.76	0.18
Age, years, mean (SD)	22.6 (4.0)	21.3 (5.4)	21.1 (5.1)	20.6 (4.0)	*F*(1,210) = 3.69	0.06	*F*(1,89) = 0.97	0.76
Education, years, mean (SD)^a^	15.0 (2.3)	12.4 (2.5)	12.3 (2.6)	12.0 (1.7)	*F*(1,208) = 59.88	<0.001	*F*(1,88) = 0.17	0.68
Parental education, years, mean (SD)^b^	14.0 (2.3)	13.8 (2.5)	13.8 (2.7)	14.0 (1.9)	*F*(1,177) = 0.35	0.56	*F*(1,78) = 0.06	0.81
Handedness, right/both/left, no.^c^	88/0/1	83/6/16	60/4/12	10/2/1	*χ*^2^(2) = 18.19	<0.001	*χ*^2^(2) = 2.19	0.33
Antipsychotic medication dose, Chlorpromazine equivalent mg/day, mean (SD)^d^		181.5 (143.3) [*n* = 43]	174.3 (113.8) [*n* = 29]	161.7 (127.0) [*n* = 6]			*F*(1,34) = 0.06	0.81
Antipsychotic medication type, typical/atypical/mixed, no.^e^		5/36/2	5/23/1	0/5/1			*χ*^2^(2) = 2.57	0.28
Antidepressant medication dose, Imipramine equivalent mg/day, mean (SD)^f^		88.1 (45.5) [*n* = 25]	81.3 (42.4) [*n* = 18]	105.4 (52.0) [*n* = 7]			*F*(1,24) = 1.43	0.24
GAF score at baseline, mean (SD)^g^		49.6 (10.1)	48.7 (8.4)	51.7 (13.0)			*F*(1,89) = 1.18	0.28
GAF score at 1-year follow-up, mean (SD)^h^		58.1 (13.2)	60.6 (11.6)	43.2 (12.4)			*F*(1,89) = 24.65	<0.001
CAARMS subscores^i^								
Unusual thought global rating scale, mean (SD)		3.6 (1.3)	3.8 (1.3)	2.5 (1.4)			*F*(1,49) = 4.84	0.03
Unusual thought frequency scale, mean (SD)		4.4 (1.5)	4.5 (1.4)	3.7 (2.0)			*F*(1,49) = 1.84	0.18
Perceptual abnormalities global rating scale, mean (SD)		2.9 (1.6)	2.8 (1.6)	3.3 (0.5)			*F*(1,49) = 0.69	0.41
Perceptual abnormalities frequency scale, mean (SD)		2.9 (1.7)	3.0 (1.8)	3.2 (1.2)			*F*(1,49) = 0.08	0.78
Disorganized speech global rating scale, mean (SD)		2.0 (1.2)	2.0 (1.3)	1.8 (1.5)			*F*(1,49) = 0.15	0.71
Disorganized speech frequency scale, mean (SD)		3.9 (2.2)	3.9 (2.2)	4.3 (2.3)			*F*(1,49) = 0.23	0.63
SIPS/SOPS subscores								
Unusual thought content/delusional ideas, mean (SD)		3.5 (1.8)	3.6 (1.9)	3.3 (2.1)			*F*(1,38) = 0.12	0.73
Suspiciousness/persecutory ideas, mean (SD)		3.3 (1.5)	3.3 (1.5)	3.1 (1.8)			*F*(1,38) = 0.05	0.83
Grandiose ideas, mean (SD)		1.0 (1.3)	1.3 (1.4)	0.6 (1.1)			*F*(1,38) = 1.48	0.23
Perceptual abnormalities/hallucinations, mean (SD)		3.2 (1.9)	3.1 (2.1)	2.9 (2.0)			*F*(1,38) = 0.08	0.79
Disorganized communication, mean (SD)		2.3 (1.9)	2.7 (1.9)	2.0 (1.4)			*F*(1,38) = 0.91	0.35
BACS subscores^j^								
List learning, mean (SD)		50.2 (9.8)	49.9 (10.8)	51.8 (6.4)			*F*(1,62) = 0.31	0.58
Digit sequencing task, mean (SD)		20.1 (5.1)	19.7 (5.4)	21.5 (4.4)			*F*(1,62) = 1.06	0.31
Token motor task, mean (SD)		71.6 (14.5)	71.4 (15.3)	72.6 (12.2)			*F*(1,62) = 0.06	0.81
Category and letter fluency, mean (SD)		44.7 (13.2)	44.7 (14.7)	42.5 (9.0)			*F*(1,62) = 0.23	0.63
Tower of London task, mean (SD)		18.1 (2.3)	18.1 (2.3)	18.2 (2.9)			*F*(1,62) = 0.003	0.95
Symbol coding, mean (SD)		68.1 (15.4)	69.6 (16.5)	65.4 (11.4)			*F*(1,62) = 0.64	0.43
Intracranial volume, cm^3^, mean (SD)	1548.5 (139.4)	1537.6 (162.4)	1549.0 (159.7)	1515.9 (175.2)	*F*(1,210) = 0.43	0.51^k^	*F*(1,89) = 0.49	0.49^k^

*ARMS* at-risk mental state, *ARMS-NR* non-resilient ARMS individuals, *ARMS-P* ARMS who subsequently developed psychosis, *ARMS-R* resilient ARMS individuals, *BACS* Japanese version of the Brief Assessment of Cognition in Schizophrenia, *CAARMS* comprehensive assessment of at-risk mental states, *GAF* Global Assessment of Functioning, *HC* healthy controls, *SIPS/SOPS* the structured interview for prodromal symptoms/the scale of prodromal symptoms.

^a^Data missing for 2 individuals.

^b^Data missing for 33 individuals.

^c^Data missing for 17 individuals.

^d^Different typical and atypical antipsychotic dosages were converted into Chlorpromazine equivalents using the guideline by Inada and Inagaki [37].

^e^43 individuals received medication therapy.

^f^Different antidepressant dosages were converted into Imipramine equivalents using the guideline by Inada and Inagaki [37].

^g^Data missing for 1 individual.

^h^Data missing for 17 individuals.

^i^Data missing for 2 individuals.

^j^Data missing for 37 individuals.

^k^Analysis of covariance with age as a covariate was used for group comparison.

### Image acquisition and processing

The subjects underwent 1.5- (Toyama, Toho, and Tohoku) and 3-Tesla (Tokyo) MRI using the protocols at each site (Supplementary Table 1). Obtained T1-weighted MR scans were preprocessed by FreeSurfer software (ver.5.3.) [44]. The implemented processing stream was involved in removal of non-brain tissue, transformation to Talairach-like space, segmentation of gray and white matter tissue, triangular tessellateion, and inspection of the white matter and pial surfaces. One trained researcher (DS) who was blinded to the subjects’ identities visually inspected the reconstructed images and manually corrected any errors of the cortical segmentations. Each vertex-wise CT value, which was computed as the minimum distance between pial and white surfaces for each vertex, was mapped on a common spherical coordinate system smoothed with a 10-mm full width at half-maximum Gaussian kernel.

### Statistical analysis

Statistical testing for demographic differences among groups was conducted by one-way ANOVA or chi-square tests.

Potential contribution of baseline clinical variables (positive symptoms subscale scores of the CAARMS [*n* = 50] or SOPS [*n* = 39] and BACS subscores [*n* = 63]) to functional outcomes (i.e., follow-up GAF score at 1 year) was tested using univariate linear regression analyses in SPSS (ver. 22.0.0) (IBM Corp., Armonk, NY). The significance threshold was set at *p* < 0.05 (two-tailed) and a Bonferroni correction was applied to prevent a possible type I error due to multiple tests.

Within FreeSurfer’s Query Design Estimate Contrast application, a general linear model controlling for age, sex, and scanning sites was used to estimate group differences in the CT value at each vertex. Vertex-by-vertex whole-brain CT correlation analyses with baseline clinical variables (duration of education [*n* = 209], antipsychotic medication dose [*n* = 43], positive symptoms subscale scores of the CAARMS [*n* = 55] or SOPS [*n* = 50], and BACS subscores [*n* = 70]) were also carried out by a general linear model controlling for age, sex, and scanning sites. A Monte Carlo Simulation, which was embedded in the Analysis of Functional NeuroImages’ AlphaSim program (NIMH, Bethesda, MD, USA), was applied to correct for multiple comparisons [45]. To define significant clusters, 10,000 iterations of simulations were employed for each comparison whose significance threshold was set at *p* < 0.05 (two-tailed and corrected for multiple testing).

In order to overcome the problem of inter-site bias, we conducted two corroborative analyses. First, we adapted a prospective meta-analysis approach similar to recent multisite studies [46, 47] using SPSS (ver. 22.0.0) and Review Manager (ver. 5.3) (the Nordic Cochrane Centre, Cochrane Collaboration, Copenhagen, Denmark). Each CT value was extracted from ten regions of interest (ROIs), where significant group differences in CT were detected (Table 2). Group differences in CT in the ROIs were investigated by each site and Cohen’s *d* effect sizes were calculated from the group contrasts. Those for each site were entered into a random-effect model meta-analysis and overall effect sizes were obtained. Second, we employed a Combat algorithm, which was a novel and reliable harmonization technique for combining brain measures across multisite [48, 49], to remove unwanted scanner variations. The harmonized CT values in the ten ROIs (Table 2) were compared across the groups using ANCOVA with age and sex as covariates. We also performed an unsupervised dimension reduction of the CT measurement using principal component analysis to visualize how the variation in the data was related to scanning site before and after the harmonization.

**Table 2:** Clusters with significant group differences in cortical thickness.

Cluster no.	Location of peak vertex (cluster name)	MNI coordinates			Cluster size (mm^2^)	Cluster-wise probability *P* value
		*x*	*y*	*z*		
ARMS < HC						
No. 1	Left superior temporal	−50.1	−19.4	−5.3	4612.83	0.0001
No. 2	Left frontal pole	−8.6	61.3	−9.2	4448.44	0.0001
No. 3	Left insula	−27.7	22.9	4.3	1243.00	0.0028
No. 4	Right fusiform	36.7	−39.6	−10.7	4906.34	0.0001
No. 5	Right superior frontal	7.9	56.9	18.8	1977.49	0.0001
No. 6	Right precuneus	10.2	−46.9	28.8	869.66	0.0419
ARMS > HC						
No. 7	Left postcentral	−19.9	−28.8	58.5	1118.75	0.0056
No. 8	Left precentral	−42.0	−9.0	31.6	932.70	0.0198
No. 9	Right pericalcarine	17.9	−71.3	11.3	1097.77	0.0088
ARMS-NR < ARMS-R						
No. 10	Right postcentral	14.1	−33.5	70.0	952.96	0.0233

*ARMS* at-risk mental state, *ARMS-NR* non-resilient ARMS individuals, *ARMS-R* resilient ARMS individuals, *HC* healthy controls, *MNI* Montreal Neurological Institute.

Given the previously reported findings of larger cortical surface area (SA) for ARMS-R compared to ARMS-NR [34], we also conducted group comparisons in the SA values using a similar general linear model.

## Results

### Demographic background

Groups were matched for age, sex, and parental education, but the healthy controls were characterized by higher education and higher rate of dextrality than the whole ARMS group (Table 1). The ARMS-R and -NR groups did not differ in baseline GAF score, medication status, rate of developing psychosis, *χ*^2^(1) = 0.17, *p* = 0.68, or BACS subscores, but the ARMS-NR individuals exhibited fewer severe unusual thoughts at baseline and lower GAF score at follow-up than the ARMS-R individuals (Table 1).

### Baseline clinical variables and outcome

Univariate linear regression analyses of ARMS subsamples demonstrated no significant contribution of baseline CAARMS (*n* = 50), SOPS (*n* = 39), or BACS (*n* = 63) subscores to follow-up GAF scores (Supplementary Table 2).

### Group comparison of CT

**Fig. 1:**
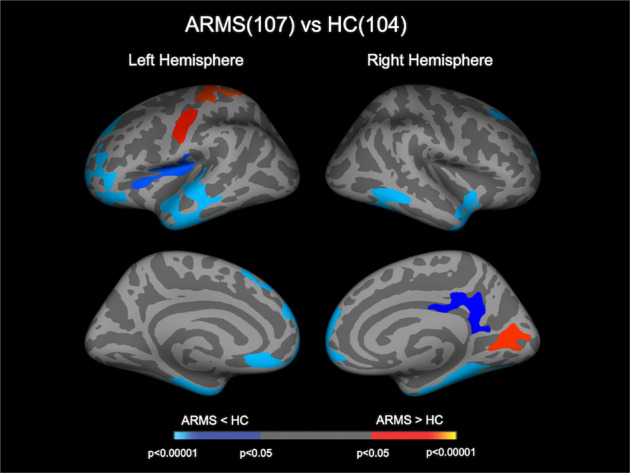
Clusters showing differences in CT between ARMS individuals and HC. Cortical statistical maps displaying altered CT in individuals with ARMS compared with HC. The maps are shown for the right and left hemispheres in lateral and medial views, respectively. The horizontal bar shows *p* values corrected for multiple comparisons. ARMS at-risk mental state, CT cortical thickness, HC healthy control.

Compared with the controls, the ARMS group as a whole exhibited a significantly reduced CT in the frontal pole, superior frontal gyri, orbitofrontal cortices, entorhinal cortices, parahippocampal gyri, fusiform gyri, lateral temporal gyri, temporal pole, and insular cortices bilaterally, and in the left middle frontal gyrus, right posterior and isthmus cingulate gyri, and right precuneus cortex. They also exhibited a significantly increased CT in the left pre- and postcentral gyri, right lingual gyrus, and right pericalcarine cortex (Fig. 1 and Table 2).

There were no significant differences in CT between the ARMS individuals who later developed psychosis (ARMS-P) and those who did not (ARMS-NP) at baseline.

**Fig. 2:**
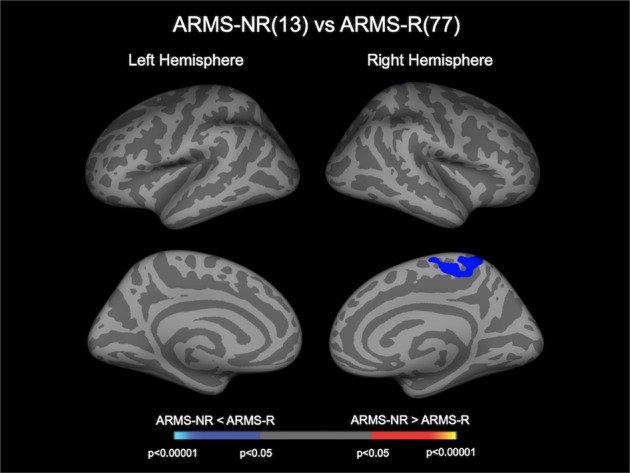
Cluster showing difference in CT between ARMS-R and ARMS-NR. Cortical statistical maps displaying reduced CT in the ARMS-NR group compared with the ARMS-R group. The horizontal bar shows *p* values corrected for multiple comparisons. ARMS at-risk mental state, ARMS-NR non-resilient ARMS individuals, ARMS-R resilient ARMS individuals, CT cortical thickness.

The ARMS-NR group exhibited a significantly reduced CT in the right paracentral lobule compared with the ARMS-R group (Fig. 2 and Table 2). This cluster remained significant even when we strictly defined the ARMS-NR (baseline GAF score > follow-up score and follow-up score < 65, *n* = 12) and ARMS-R (baseline score ≤ follow-up score and follow-up score ≥ 65, *n* = 31) subgroups according to previous reports [30] (Supplementary Fig. 1). As supplementary analyses, we performed group comparisons (e.g., ARMS-R vs. controls, ARMS-NR vs. controls, and ARMS-P vs. ARMS-NP) in the mean CT values of the cluster in the right paracentral lobule as well as correlation analyses between those and behavioral or clinical variables (duration of education, antipsychotic medication dosage, CAARMS or SOPS, and BACS subscores); although there were no significant differences between ARMS-R and controls or between ARMS-P and ARMS-NP, ARMS-NR exhibited significantly lower CT values compared to controls (Supplementary Tables 3–5). No significant relationships were found between the CT value in this extracted cluster and behavioral or clinical variables. There were no regions where the ARMS-NR group exhibited a significantly increased CT compared with the ARMS-R group.

Supplementary analyses using meta-analytic overall effect sizes and Combat harmonization demonstrated no major effects of scanning site on the CT findings in this study (Supplementary Tables 6, 7 and Supplementary Figs. 2, 3). There was no obvious clustering by scanning site even in the raw data points (with a few exceptions in Toho University), and the harmonized data points were equally distributed across sites (Supplementary Fig. 4).

### Relationship of CT with cognitive measures and clinical variables

**Fig. 3:**
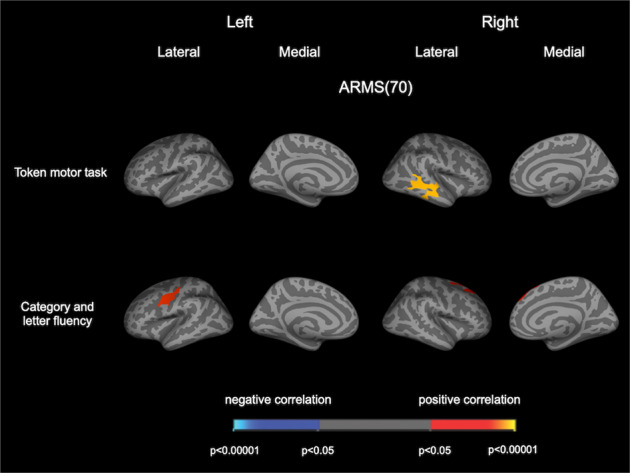
Cortical statistical maps displaying the relationship between CT and cognitive performance in the ARMS group. Cortical statistical maps displaying the relationship between CT and BACS subscores (token motor task and verbal fluency) in individuals with ARMS. The horizontal bar shows *p* values corrected for multiple comparisons. ARMS at-risk mental state, BACS Japanese version of the Brief Assessment of Cognition in Schizophrenia, CT cortical thickness.

For the BACS subscores, the Token motor task scores were positively correlated with CT in the right superior and middle temporal gyrus, and the Category and letter fluency scores were positively correlated with CT in the left precentral gyrus and right superior frontal gyrus in the entire ARMS population (Fig. 3). The other BACS subscores, antipsychotic medication dosage, CAARMS or SOPS subscores, or duration of education were not associated with CT in any region.

### Group comparison of SA

There were no significant differences in SA between the ARMS and controls or between ARMS-R and ARMS-NR.

## Discussion

This is, to the best of our knowledge, the first multicenter MRI study to investigate the association between CT abnormalities and subsequent functional outcomes in a relatively large clinical high-risk cohort. ARMS individuals as a whole had a significantly reduced CT, mainly in the fronto-temporal areas, and an increased CT in the parieto-occipital areas, which were partially associated with cognitive functioning. The ARMS-NR group had a significantly reduced CT of the right paracentral lobule compared with the ARMS-R group, which may be a predictor of the short-term functional outcome irrespective of the manifestation of overt psychotic symptoms.

Reduced fronto-temporal CT in our ARMS cohort, which was similar to CT findings in schizophrenia [22], was consistent with previous high-risk studies [31, 32, 50], albeit with negative findings in other ARMS cohorts [33, 51]. Reduced CT may be prominent, particularly in the ARMS-P group [52–55], but the present and previous studies [32, 51, 56] found no CT difference between the ARMS-P and -NP groups at baseline, although one of those studies showed progressive regional cortical thinning in the ARMS-P group [51]. These discrepancies may be partly due to the heterogeneity of the ARMS cohorts; our cohort was characterized by severe subthreshold symptomatology relative to those with negative CT findings [33, 51]. It should also be noted that fewer ARMS-P subjects (*n* = 3/21 [14.3%]) received antidepressants, which can induce cortical thinning [57], than ARMS-NP subjects (*n* = 20/72 [27.8%]). Consistent with several studies on schizophrenia and ARMS [58, 59], we also noted increased CT in the left parietal and right occipital regions in the ARMS group relative to the controls. Parietal and occipital cortices may be comparatively preserved from progressive pathological processes [60, 61] because cortical maturation of these regions is mostly completed by the time of psychosis onset [62]. Taken together, our study supports ARMS subjects sharing bidirectional alterations of CT with patients with overt schizophrenia, which may reflect a complicated neurodevelopmental misbalance and its compensatory change across different brain areas [59].

The most important finding in this study was that a reduced CT of the right paracentral lobule distinguished the ARMS-NR subgroup from the ARMS-R subgroup. Although there were methodological differences (e.g., definition of “resilience”, surface-based ROI analysis vs. vertex-wise analysis), the present study replicated the findings by de Wit et al. [34] in demonstrating a reduced CT specific to the non-resilient ARMS group, which may be due to disturbed compensatory neural mechanisms [34]. Although they found broad CT differences in the frontal and temporo-parietal regions between resilient and non-resilient individuals, our study suggested a specific role of the paracentral lobule in functional outcomes in the ARMS population. A resting-state functional MRI study in healthy subjects revealed that the paracentral lobule is functionally connected to other frontal and parietal regions, subserving motor functioning and spatial attention [63]. Of note, previous studies on schizophrenia demonstrated that some components of motor abnormalities (e.g., gesture deficits and neurological soft signs) and attention impairment [64], which can be caused by paracentral deficits [65] and be associated with social deficits [66, 67], predicted poor 1-year functional outcomes [68]. Although the present study suggested an altered CT of the paracentral lobule to be a potential predictor of functional decline, further studies of more detailed motor-related abnormalities and disturbed attention subdomain are required to assess whether it can predict functional outcomes in high-risk cohorts.

In the ARMS individuals who underwent BACS, we found that CT reduction in the superior and middle temporal gyrus, and that in the precentral gyrus and superior frontal gyrus are associated with poor motor speed and impaired verbal fluency, respectively. The results were partly similar to the associations between gray matter volume and cognitive functioning in schizophrenia patients [69, 70] and ARMS-P individuals [71, 72], supporting the existence of cognitive deficits even before psychosis onset as a trait vulnerability marker [73]. Baseline cognition, particularly in verbal fluency and verbal memory in ARMS individuals, was also reported to predict short- (i.e., 1-year follow-up) [74, 75] and medium to long-term (~3–8 years) [64, 75, 76] functional outcomes. Although we failed to detect a significant relationship between the baseline BACS score and functional outcome in a subsample of our ARMS cohort (*n* = 63), future studies with a larger cohort should further examine the potential contribution of baseline cognition and its neuronal underpinnings to prediction of the clinical course of high-risk individuals.

This study has several limitations. First, although we processed all MR images similarly and included the scanning sites as covariates in the statistical model, the effects of scanner differences (Supplementary Table 1) may have confounded the findings. However, the current “applied” meta-analytic method and harmonization technique can better manage inter-scanner variances and largely replicated our main results (Supplementary Tables 6, 7 and Supplementary Figs. 2, 3). Second, we were unable to exclude potential confounding effects from antipsychotic medication. However, the antipsychotic medication dosage was not associated with CT in this ARMS cohort. As preceding studies reported altered CT in medication-naive subjects of early psychosis [77, 78], our main findings may not be explained solely by antipsychotic medication. Lastly, a few previous studies have examined the relationship between brain structural/functional abnormalities and subsequent functional outcomes in ARMS individuals, and their definitions of functional outcome subgroups have been inconsistent [34, 79]. While we used a novel definition to classify ARMS-R and -NR, the CT difference we found was replicated in the subanalyses using a more stringent definition that combined our definition with that in the previous study [34].

In conclusion, this multisite study demonstrated that ARMS individuals are characterized by cortical thinning in the fronto-temporal regions and cortical thickening in the parieto-occipital regions, which may reflect general vulnerability to psychopathology. In addition, fronto-temporal cortical thinning may reflect neural underpinnings of cognitive impairments at the prodromal stage of psychosis. Moreover, cortical thinning of the right paracentral lobule in high-risk subjects may be associated with functional decline as a possible prognostic predictor.

## Supplementary information

Supplementary Information
