# Supplementary material for: Reduced cortical thickness of the paracentral lobule in at-risk mental state individuals with poor 1-year functional outcomes
Source: Transl Psychiatry. 2021 Jul 14;11:396. doi: 10.1038/s41398-021-01516-2 (PMC8289863; doi:10.1038/s41398-021-01516-2)
Supplement: Supplementary file 1 — Supplementary Information [file 41398_2021_1516_MOESM1_ESM.docx]

**Supplementary Information**

**Supplementary Table 1.** Magnetic resonance imaging protocols for 4 scanning sites

**Supplementary Table 2.** Univariate linear regression analyses of the determinants associated with BACS, CAARMS, and SOPS subscores

**Supplementary Table 3.** Group comparison of the cortical thickness values between the ARMS-R and controls

**Supplementary Table 4.** Group comparison of the cortical thickness values between the ARMS-NR and controls

**Supplementary Table 5.** Group comparison of the cortical thickness values between the ARMS-P and ARMS-NP

**Supplementary Table 6.** Group comparison of the harmonized cortical thickness values between the ARMS and controls

**Supplementary Table 7.** Group comparison of the harmonized cortical thickness values between the ARMS-R and ARMS-NR

**Supplementary Figure 1.** Group comparison of cortical thickness between strictly defined ARMS-R and ARMS-NR

**Supplementary Figure 2a-i.** The forest plots for the clusters that showed significant group differences between individuals with ARMS and healthy controls

**Supplementary Figure 3.** The forest plots for the clusters that showed significant group differences between ARMS-R and ARMS-NR

**Supplementary Figure 4.** Unsupervised dimension reductions before and after combat harmonization

**Supplementary Figure 1.** Group comparison of cortical thickness between strictly defined ARMS-R and ARMS-NR


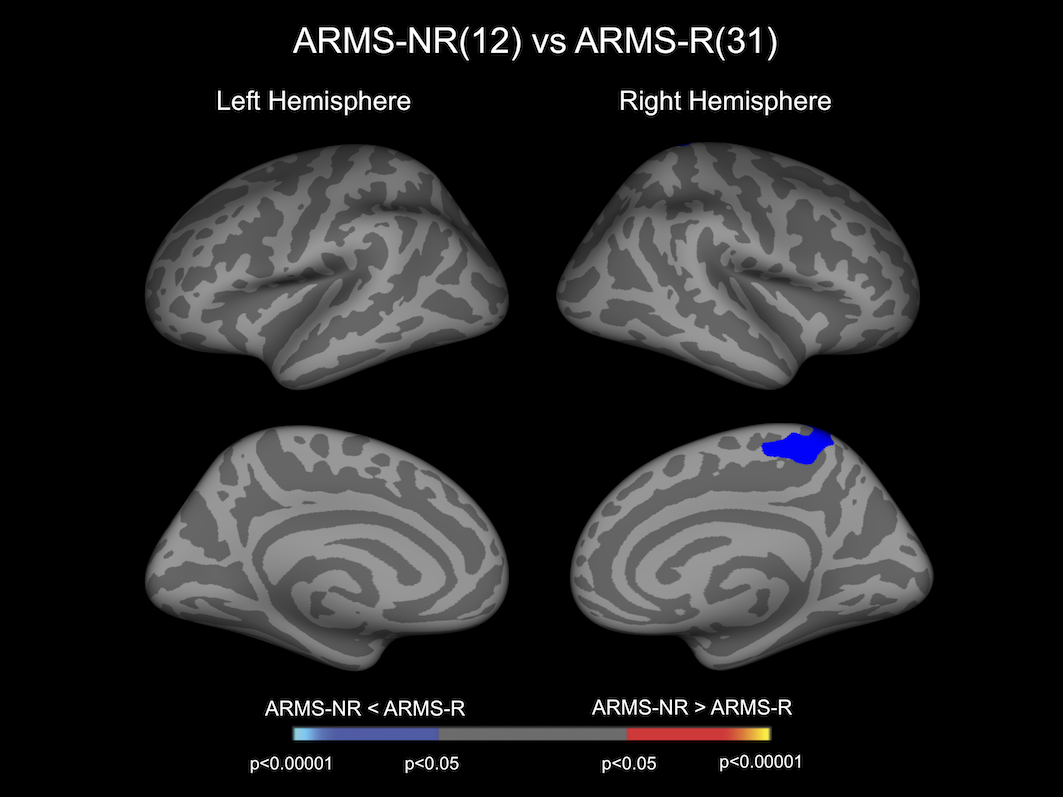


Cortical statistical maps displaying reduced cortical thickness in the strictly defined ARMS-NR group (*n* = 12) compared with the strictly defined ARMS-R group (*n* = 31) controlling for age, sex, and scanning sites. The significance threshold was set at *p* < 0.05 (two-tailed and corrected by a Monte Carlo Simulation). Horizontal bar shows *p*-values corrected for multiple comparisons.

**Supplementary Figure 2.** The forest plots for the clusters that showed significant group differences between individuals with ARMS and healthy controls

Cohen’s *d* effect sizes ± standard errors for regional cortical thickness differences between individuals with ARMS and healthy controls. Effect sizes were corrected for sex and age. The forest plots for the clusters with significant group differences between individuals with ARMS and healthy controls are shown in Supplemental Figures S2a-i [(a) Cluster No.1, left superior temporal, (b) Cluster No.2, left frontal pole, (c) Cluster No.3, left insula, (d) Cluster No.4, right fusiform, (e) Cluster No.5, right superior frontal, (f) Cluster No.6, right precuneus, (g) Cluster No.7, left postcentral, (h) Cluster No.8, left precentral, and (i) Cluster No.9, right pericalcarine].

Abbreviations: ARMS, at-risk mental state

**Supplementary Figure 2a**

Cluster No.1, left superior temporal

**Supplementary Figure 2b**

Cluster No.2, left frontal pole

**Supplementary Figure 2c**

Cluster No.3, left insula

**Supplementary Figure 2d**

Cluster No.4, right fusiform

**Supplementary Figure 2e**

Cluster No.5, right superior frontal

**Supplementary Figure 2f**

Cluster No.6, right precuneus

**Supplementary Figure 2g**

Cluster No.7, left postcentral

**Supplementary Figure 2h**

Cluster No.8, left precentral

**Supplementary Figure 2i**

Cluster No.9, right pericalcarine

**Supplementary Figure 3.** The forest plots for the clusters that showed significant group differences between ARMS-R and ARMS-NR

Cohen’s *d* effect sizes ± standard errors for regional cortical thickness differences between ARMS-R and ARMS-NR groups. Effect sizes were corrected for sex and age. The forest plot for the cluster with significant group differences between ARMS-R and ARMS-NR groups (Cluster No.10, right postcentral) is shown below.

Abbreviations: ARMS, at-risk mental state; ARMS-NR, non-resilient ARMS individuals; ARMS-R, resilient ARMS individuals

Cluster No.10, right postcentral

**Supplementary Figure 4.** Unsupervised dimension reductions before and after combat harmonization


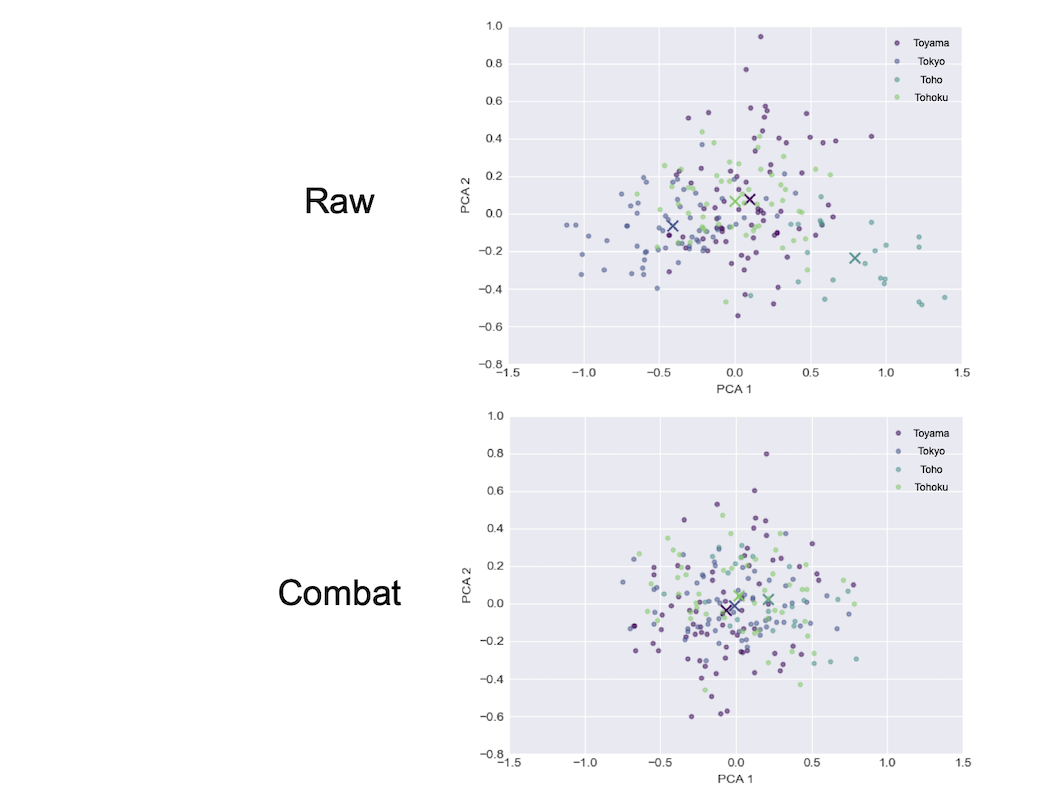


　We conducted principal component analysis (PCA) to reduce the dimension of the cortical thickness measures in an unsupervised manner. We presented the projection of the data into first two PCA coordinates. The crosshairs were located at the center of gravity of each site cluster (shown in the same color).
